# Role of Cardiac Magnetic Resonance to Improve Risk Prediction following Acute ST-elevation Myocardial Infarction

**DOI:** 10.3390/jcm9041041

**Published:** 2020-04-07

**Authors:** Martin Reindl, Ingo Eitel, Sebastian Johannes Reinstadler

**Affiliations:** 1 University Clinic of Internal Medicine III, Cardiology and Angiology, Medical University of Innsbruck, Anichstraße 35, A-6020 Innsbruck, Austria; Martin.Reindl@tirol-kliniken.at; 2 University Heart Center Lübeck, Medical Clinic II (Cardiology/Angiology/Intensive Care Medicine), University Hospital Schleswig-Holstein, Ratzeburger Allee 160, D-23538 Lübeck, Germany; Ingo.Eitel@uksh.de; 3 German Center for Cardiovascular Research (DZHK), Partner Site Hamburg/Kiel/Lübeck, D-23538 Lübeck, Germany

**Keywords:** ST-elevation myocardial infarction, cardiac magnetic resonance imaging, risk assessment, risk stratification, prognosis

## Abstract

Cardiac magnetic resonance (CMR) imaging allows comprehensive assessment of myocardial function and tissue characterization in a single examination after acute ST-elevation myocardial infarction. Markers of myocardial infarct severity determined by CMR imaging, especially infarct size and microvascular obstruction, strongly predict recurrent cardiovascular events and mortality. The prognostic information provided by a comprehensive CMR analysis is incremental to conventional risk factors including left ventricular ejection fraction. As such, CMR parameters of myocardial tissue damage are increasingly recognized for optimized risk stratification to further ameliorate the burden of recurrent cardiovascular events in this population. In this review, we provide an overview of the current impact of CMR imaging on optimized risk assessment soon after acute ST-elevation myocardial infarction.

## 1. Introduction

The discovery and subsequent adoption of novel evidence-based therapies in patients suffering from acute ST-elevation myocardial infarction (STEMI) during the last decades led to a significant improvement of survival and dramatically reduced the risk of recurrent cardiovascular events [1]. However, remaining differences in the risk for future cardiovascular events stimulates the ongoing search for optimized patient-specific prognostication soon after STEMI [2]. Cardiac magnetic resonance (CMR) imaging performed during the early phase post-STEMI has been intensively investigated for this purpose. This technique is uniquely suitable to comprehensively assess functional and morphological myocardial tissue alterations as well as post-infarct complications [3]. Cine imaging, which is an essential part of each CMR protocol, provides a detailed analysis of right and left chamber volumes and cardiac function (mainly ejection fraction) with crucial prognostic implications [4]. Importantly, cine CMR holds much more detailed information that enables assessment of more comprehensive markers of cardiac function, such as myocardial strain that may be even more informative for prognostication after STEMI than standard chamber volumes and ejection fraction [5,6,7]. A large body of evidence has demonstrated the great value of infarct severity assessment with CMR for the prediction of future cardiovascular events [3]. In this regard, late gadolinium enhancement (LGE) CMR, which is currently considered the reference method for infarct sizing, and microvascular obstruction (MVO) assessment, as a marker of severe reperfusion injury, has been increasingly used for improved long-term risk stratification and to evaluate cardioprotective interventions [3]. Additional imaging biomarkers of myocardial tissue alterations using novel CMR sequences have also been recently evaluated and may provide further insights into STEMI pathophysiology and risk prediction. Multi-parametric CMR imaging revealing cardiac function, myocardial tissue alterations, and infarct complications in a single examination has therefore an emerging role for optimized risk prediction post-STEMI and could thus be an important step forward for personalized medicine in this context. In this review, we outline the role of comprehensive tissue characterization by CMR for improved risk stratification soon after acute STEMI.

## 2. Myocardial Function

### 2.1. Left Ventricular Function

**Figure 1:**
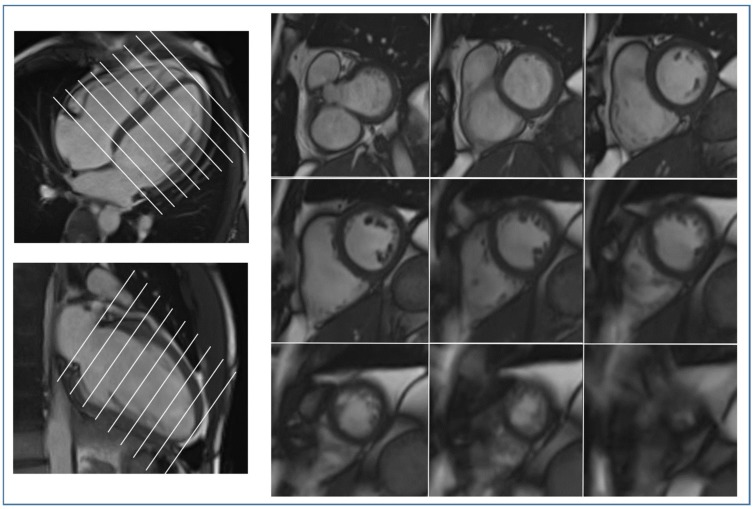
Measurement of left ventricular volumes and function. A stack of short axis images is used for semi-automated delineation of epicardial and endocardial borders on both end-systolic and end-diastolic images.

Left ventricular (LV) ejection fraction is the most widely applied parameter for the measurement of resting global LV function and its determination before hospital discharge is recommended by contemporary STEMI guidelines [8]. Due to its broad availability, echocardiography still represents the preferred modality to assess LV ejection fraction in daily clinical routine [8]. However, CMR provides superior accuracy and reproducibility than echocardiography and has become the in vivo gold-standard modality for the quantification of LV ejection fraction [9]. Steady-state free precession (SSFP) cine imaging is the recommended CMR technique for the evaluation of LV ejection fraction, based on the high signal-to-noise ratio as well as excellent contrast between myocardium and blood pool provided by this sequence [10,11]. To obtain LV ejection fraction, measurements of LV dimensions are typically performed by semi-automated delineation of epi- and endocardial borders on end-systolic and end-diastolic short axis images (Figure 1) [12]. Although standard cine CMR is well established, it requires multiple scans with breath-holds for whole-heart coverage. Several approaches have therefore been investigated to reduce the number of breath-holds as well as examination times [13,14,15]. Among them, single-breath-hold compressed sensing cine CMR has been shown to reduce scan times in daily practice while providing exact measurements of cardiac volumes and function [16,17,18]. Consequently, single-breath-hold compressed sensing techniques have the potential to replace the traditional multi-breath-hold cine CMR sequences. Deep learning image analysis algorithm might further facilitate the clinical use by providing automated and fast analysis of cardiac function from cine CMR [19].

There is a large body of evidence confirming that CMR-determined LV ejection fraction strongly determines clinical outcome after STEMI [20,21,22,23,24,25]. Indeed, the validity of LV ejection fraction for the prediction of hard clinical events post-STEMI was shown to be independent and incremental to established outcome markers including LV ejection fraction by echocardiography [20,21,23]. At the same time, LV ejection fraction is limited by reflecting only global systolic function, whereas regional functional abnormalities remain insufficiently depicted [26,27]. Myocardial strain analysis evaluates deformation of the myocardium throughout the cardiac cycle, allowing determination of both global and regional LV function [26,27]. Over the last decade, this more comprehensive approach of LV function assessment has become available for CMR [27,28,29]. Several CMR techniques for strain analysis including myocardial tagging, strain-encoding imaging, phase-contrast imaging, and cine-derived strain imaging have been developed [5]. Due to its retrospective nature without the need for additional breath-hold scans, cine-derived strain imaging (feature-tracking and tissue-tracking) has emerged as predominant approach used in recent STEMI studies [5]. As shown by several investigations, cine-derived strain analyses can be performed with high accuracy and reproducibility after STEMI [5,30,31]. Moreover, a good correlation with infarct severity has been demonstrated [31]. All global strain parameters (global longitudinal, radial, and circumferential strain) derived from early CMR imaging after STEMI were shown to be of prognostic value in this setting [32,33]. In particular, global longitudinal strain was demonstrated to provide strong validity for the prediction of major adverse cardiovascular events (MACE) post-STEMI, incremental to LV ejection fraction and infarct severity markers [32]. Although the explanation for the crucial prognostic relevance of global longitudinal strain after infarction has not been entirely elucidated, this link most probably is based on the primarily longitudinal orientation of subendocardial myocardial fibers [26]. In light of the “wavefront phenomenon” describing that ischemic myocardial injury starts at subendocardial layers [34], the longitudinal contraction is predominantly affected in the setting of revascularized STEMI, which would explain the high sensitivity and strong prognostic value of global longitudinal strain in this patient population [26]. In agreement with this pathophysiological concept, recent data by Schuster et al. [35] and Mayr et al. [36] revealed that other, more practicable and simpler CMR parameters of LV longitudinal function including manual long axis strain and mitral annular plane systolic excursion also provide prognostic information post-STEMI above LV ejection fraction. In contrast, other integrative approaches for cardiac function assessment, such as the LV global function index, have been studied but added limited or no additional information over LV ejection fraction [37,38,39]. Together, new CMR-based applications of comprehensive LV function evaluation have increased our understanding of global and regional systolic dysfunction post-STEMI; however, further studies are needed to find the best integrative functional approach and to define accepted cut-off values of these new measures to enable optimized risk stratification in clinical practice.

### 2.2. Right Ventricular Function

In comparison to the left ventricle, CMR data on the clinical and prognostic validity of right ventricular (RV) function in acute STEMI are scarce. The “forgotten” right chamber, however, may be affected in the setting of acute STEMI by direct infarction per se, myocardial stunning and as a subsequent result of LV heart failure [40]. RV function is most frequently quantified via RV ejection fraction, which is measured by CMR in the same manner as LV ejection fraction using cine SSFP [12]. The prognostic implications of CMR-determined RV function in STEMI patients undergoing contemporary percutaneous coronary intervention (PCI) are not completely clarified. The study by Miszalski-Jamka et al. including 99 STEMI patients demonstrated an independent association between RV ejection fraction and MACE, even after adjustment for RV myocardial injury and LV ejection fraction [41]. However, in the larger study by Grothoff and colleagues including 421 STEMI patients, the relatively weak relation of RV ejection fraction with MACE did not remain significant when adjusting for RV injury, LV ejection fraction and TIMI risk score [22]. These data indicate that RV injury is more relevant for prognosis prediction than RV ejection fraction; however, further studies in the acute setting of STEMI are needed to confirm these findings and to gain a better understanding of RV involvement. In this context, it is relevant to emphasize the study by Masci et al. revealing that RV involvement is not limited to inferior wall infarctions but was also commonly found in patients with anterior wall infarction (detection of RV edema in 33%) [42]. Furthermore, this analysis demonstrated that in most patients RV injury and dysfunction dissolve within four months post-infarction, highlighting a high potential for reversibility of acute RV dysfunction [42]. However, those patients with persisting RV dysfunction at a chronic stage after infarction are at high risk for adverse clinical events in the long term [43].

However, because of the complex shape of the right ventricle, assessment of RV function has remained challenging. Myocardial strain imaging offers great potential also for a highly accurate determination of RV function. Previous studies could already highlight the promising prognostic role of RV strain parameters in different cardiac diseases [44,45]; however, data on the prognostic significance of RV strain measures in acute STEMI are lacking thus far.

### 2.3. Atrial Function

Changes in atrial structure and function have increasingly moved into the focus of cardiovascular research, leading to the introduction of the term “atrial cardiomyopathy” in the literature [46]. The high spatial and temporal resolution of CMR particularly qualifies this modality also for a precise evaluation of atrial volumes and function. In the setting of acute STEMI, Lønborg et al. were the first to comprehensively evaluate the prognostic implications of left atrial (LA) parameters as assessed by cine CMR [47]. In a cohort of 199 patients, they disclosed LA fractional change, a LA function measure of “total emptying” of the LA, as an independent predictor of MACE [47]. In this study, LA fractional change seemed to be more prognostic than LA ejection fraction, reflecting only the “active emptying” of the LA. The larger analysis by Ledwoch and colleagues including 684 STEMI patients measured LA function only by ejection fraction and could find an independent association between LA ejection fraction and adverse clinical events; however, the prognostic value of LA ejection fraction was not incremental to LV ejection fraction [48]. Hence, more comprehensive approaches of LA function assessment have been developed and tested [49,50]. Comparable with the LV, LA strain analysis has become available for CMR enabling a comprehensive evaluation of the complex function of the LA, comprising reservoir function, conduit function and contractile booster pump function [49]. In a large multicenter CMR study including 1046 patients with STEMI and non-STEMI, Schuster et al. evaluated the prognostic implications of LA function parameters as determined by feature-tracking (LA reservoir function peak systolic strain, LA conduit strain, and LA booster pump function active strain) [50]. All three atrial strain measures showed strong associations with MACE, whereas only LA peak reservoir strain emerged as an independent and incremental prognosis marker, above and beyond LV ejection fraction, infarct severity markers, and even LV global longitudinal strain [50]. These findings suggest that an integrative CMR approach incorporating upcoming parameters such as LV global longitudinal strain and LA reservoir strain, which can be interpreted as indicator of the atrial compensatory capacity, may distinctly optimize post-STEMI risk assessment. However, further CMR studies are needed to better understand the significance of these measures of LA function and to establish them in clinical decision-making processes. CMR studies addressing the prognostic relevance of right atrial measures in acute STEMI are lacking thus far, representing a remaining research field for future investigations.

## 3. Infarct Size

**Figure 2:**
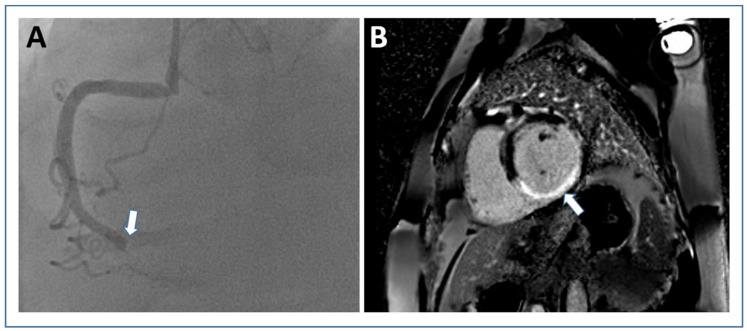
Infarct size assessment by late gadolinium-enhanced imaging. Short axis PSIR late gadolinium enhancement image ((**B**) white arrow shows “hyper-enhanced” infarct area) from a patient with transmural inferior wall infarction due to occlusion of the right coronary artery (white arrow in (**A**) angiography). Abbreviations: PSIR, phase sensitive inversion recovery.

From the several imaging modalities available, CMR is considered as the in vivo reference standard for the assessment of infarct size (IS) in STEMI patients [51,52,53]. Concretely, LGE CMR is the recommended approach for IS quantification, typically using phase-sensitive inversion recovery (PSIR) sequence (Figure 2) [12,53]. Gadolinium-based contrast media are extracellular agents unable to cross membranes of vital myocytes [53]. In acute myocardial infarction, myocyte rupture enables gadolinium-based agents to diffuse into the intracellular space, resulting in a “hyper-enhancement” of the infarcted territory as compared to remote, vital myocardium [53]. For visualization of acute IS, LGE images are acquired at least 10 min (typically 10–20 min) after bolus infection of the contrast agent [12]. In the very early phase following STEMI, reperfusion-related edema with an increase of the interstitial space causes overestimation of acute LGE-determined IS [54]. Accordingly, quantification of acute IS by LGE is recommended to be performed 3–7 days after infarction [54]. Acute IS quantified in this time range was shown to be highly prognostic in STEMI patients [55,56,57]. Indeed, IS measured in the acute phase following STEMI was confirmed to predict LV adverse remodeling and hard clinical events post-STEMI [56,58]. Importantly, the prognostic validity of CMR-determined acute IS was additive to established clinical risk factors including LV ejection fraction [24]. Hence, IS evaluation by LGE-CMR provides great potential to improve clinical risk prediction in STEMI patients. In this context, the interrelation between IS and infarct location has been controversial. A plethora of previous studies affirmed that patients with anterior wall STEMI are of higher risk for developing LV adverse remodeling, heart failure, and death [25,59,60,61,62]. However, it remained unclear whether worse prognosis in anterior STEMI patients is attributable to specific characteristics of the infarct location per se or just the result of the larger IS in these patients [59,63,64]. A recent study specifically addressing this question disclosed that the higher risk of cardiovascular complications in anterior STEMI patients is explained by the larger IS of this subgroup without any further contribution of infarct location per se [65]. The crucial prognostic role of IS has recently also been emphasized by the consensus document of the *Journal of the American College of Cardiology (JACC) Scientific Expert Panel* in which IS by LGE has been recommended as primary CMR endpoint measure in experimental and clinical trials [54]. The concept of IS assessment has lately been expanded to the entire myocardium by evaluating the presence and prognostic impact of multiple myocardial scars in the clinical setting of STEMI. Ekström et al. investigated 704 STEMI patients and revealed presence of multiple scars in approximately 8%, caused by multiple culprit lesions, procedure-related infraction due to non-culprit interventions or, predominantly, prior infarctions (silent or unknown) [66]. Moreover, it was shown that multiple myocardial scars as detected by LGE predict adverse clinical outcome (all-cause mortality and hospitalization for heart failure) independent of culprit IS and clinical prognosis markers [66]. Hence, determination of multiple scars, which might be interpreted as hard evidence of severe coronary artery disease, may serve as further risk stratification tool on top of culprit IS measurement in STEMI survivors.

Compared with LV injury analysis, evaluation of LGE in the RV myocardium is much more challenging and time-consuming due to the thin RV myocardial walls. Although RV involvement in the setting of STEMI can be reliably assessed by LGE-CMR, studies on the prognostic significance of RV myocardial injury are relatively scarce. As mentioned above, Miszalski-Jamka et al. [41] and Grothoff et al. [22] demonstrated RV infarction as strong prognosticator in STEMI patients; however, further studies are needed to validate these findings in the context of multi-parametric CMR imaging.

Parametric mapping methods (native T1 and extracellular volume mapping) have been suggested as alternatives to LGE in detecting and measuring acute IS. Indeed, Garg et al. demonstrated reliable determination of IS by parametric mapping, even indicating superior diagnostic accuracy of extracellular volume mapping as compared to LGE, which is limited by an overestimation of IS in the acute setting post-STEMI [67]. These results could be corroborated by Bulluck et al. [68]; however, these small studies could not validate their findings for hard clinical events, which remains to be done by future studies.

Compressed sensing has not only been investigated for accelerating cine CMR imaging but also shortening scan time of LGE imaging [69]. Moreover, data on an accelerated, free-breathing 3D T1 mapping technique for contrast-free myocardial tissue characterization have been published recently [70]. Despite the promising data on compressed sensing, there remain relevant drawbacks with this technique (e.g., robustness and time of the reconstruction). Whether machine learning techniques can provide a solution for a fast and efficient reconstruction of newly acquired data is intensively studied now [69].

Infarct scar entropy was recently proposed as a novel LGE marker of tissue inhomogeneity in myocardial infarction patients. Androulakis et al. analyzed a cohort of 154 myocardial infarction patients undergoing CMR prior to implantable cardioverter-defibrillator implantation [71]. The investigators found that high entropy within the infarct scar was related with ventricular arrhythmias. Moreover, high entropy of the whole left ventricle was related with mortality. Further work is warranted to investigate the exact role of infarct scar and LV entropy by LGE in the setting of STEMI.

## 4. Myocardial Edema and Salvage

**Figure 3:**
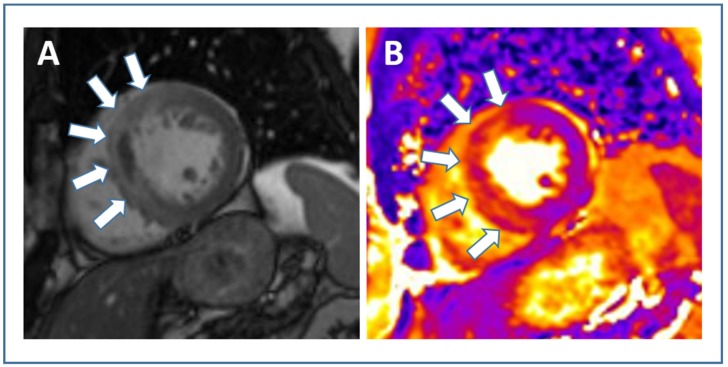
Cardiac magnetic resonance approaches to estimate myocardial area-at-risk in acute STEMI. In a case example of a STEMI patient with anterior wall infarction, area at risk is illustrated by contrast-enhanced cine SSFP ((**A**) white arrows) and T2-mapping ((**B**) white arrows). Abbreviations: STEMI, ST-elevation myocardial infarction; SSFP, steady-state free precession.

Coronary occlusion in acute STEMI leads to time-dependent cell death starting from the subendocardium and proceeding towards subepicardial layers, a process called “wavefront phenomenon” [34]. The myocardial area supplied by the occluded artery is in jeopardy to become necrotic and is therefore referred to as myocardial area at risk (AAR) [54]. The goal of timely revascularization is to terminate the progression of the necrotic wavefront and thereby prevent as much AAR as possible from becoming irreversibly damaged. Salvaged myocardium describes the part of AAR rescued by revascularization and is calculated as the difference between AAR and IS, whereas myocardial salvage index represents myocardial salvage divided by AAR [54,72]. CMR and single photon emission tomography are techniques allowing visualization of AAR and IS [72]; however, based on the higher spatial resolution and the possibility to determine both AAR and IS in a single examination, CMR has emerged as preferred imaging modality for this purpose [73]. T2-weighted edema imaging, particularly short-tau inversion recovery (STIR) sequence, is widely used to determine AAR by CMR [54,74]. As described in detail above, IS quantification is performed by using LGE CMR [73]. Although quantification of AAR and subsequently myocardial salvage using CMR has limitations (e.g., low signal-to-noise ratio, motion artifacts, incomplete blood suppression) and is still discussed controversially [75], AAR and particularly myocardial salvage index have clearly shown to predict function recovery as well as adverse remodeling and clinical events following STEMI [76,77]. Although IS represents a robust surrogate endpoint for prognosis, myocardial salvage index may be particularly preferred to evaluate therapeutic efficacy in clinical trials [74]. This is based on the fact that myocardial edema can relevantly differ also in patients with similar resulting IS [78,79]. Furthermore, in contrast to AAR and IS, myocardial salvage index is not dependent on the site of coronary occlusion [79], allowing for a standardized evaluation of treatment effectiveness. Accordingly, myocardial salvage index has frequently been used in previous large randomized clinical trials to evaluate treatment efficacy [80,81]. The optimal technique for the evaluation of AAR is still debated [3]. In consideration of the limitations of T2-weighted STIR imaging [75], more promising sequences have been introduced over the last years. Contrast-enhanced cine SSFP provides an accurate estimation of the AAR. Indeed, experimental studies found a high accuracy and precision for contrast-enhanced SSFP based AAR estimation in vivo and ex vivo [82]. In addition, AAR quantification with contrast-enhanced SSFP imaging following STEMI has a strong correlation and low bias compared with angiographic scoring [83]. A comparison between T2-STIR and contrast-enhanced cine CMR from a DANAMI 3 sub-study revealed a higher validity for contrast-enhanced cine CMR [84]. In line with this, a recent multi-vendor, multicenter comparison of contrast-enhanced SSFP and T2-STIR CMR assessing AAR after STEMI found a higher degree of diagnostic quality images with contrast-enhanced SSFP in two out of three vendors. Consequently, the authors suggested that contrast-enhanced SSFP might be more suitable for implementation in multi-vendor, multicenter trials [85]. In addition, T2 mapping has to be highlighted, as a sequence that enables imaging acquisition with superior diagnostic quality to STIR and highest reproducibility [86,87]. Edema imaging via T2 mapping has already been applied as outcome measure in clinical trials [88]; however, the value of this technique for the prediction of hard clinical events post-STEMI remains to be elucidated. A case example for AAR estimation using contrast-enhanced cine SSFP and T2-mapping in STEMI is shown in Figure 3.

## 5. Microvascular Injury

**Figure 4:**
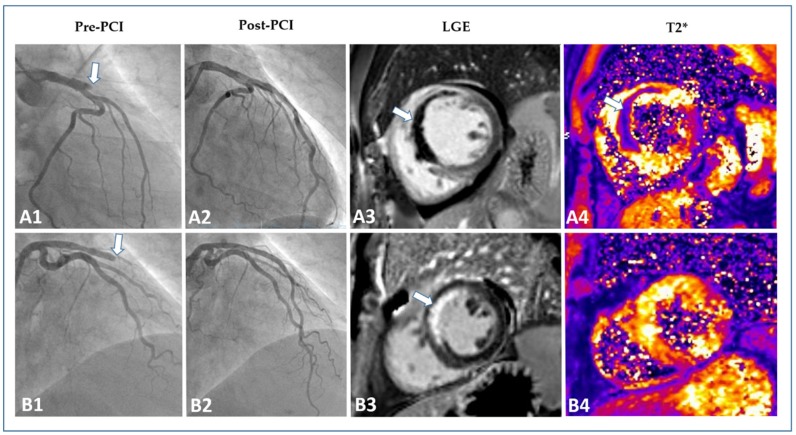
Evaluation of microvascular injury in STEMI. Two patient examples with anterior wall STEMI (occlusion of the left anterior descending artery). The first line (**A1**–**A4**) illustrates the pre-interventional (**A1**) and post-interventional angiography (**A2**) and the according CMR images ((**A3**) LGE, (**A4**) T2*) of “Patient A”. Although successfully treated by primary PCI, “Patient A” showed a large extent of microvascular obstruction (white arrow (**A3**)) and intramyocardial hemorrhage (white arrow (**A4**)). “Patient B” (second line, (**B1**–**B4**)) was also successfully treated by PCI (**B2**). This patient, in contrast, only showed hyper-enhancement without microvascular obstruction (white arrow (**B3**)) and no evidence of intramyocardial hemorrhage (**B4**). Abbreviations: STEMI, ST-elevation myocardial infarction; CMR, cardiac magnetic resonance; LGE, late gadolinium enhancement; PCI, percutaneous coronary intervention.

Development of microvascular injury, despite successful reopening of the occluded epicardial coronary artery through primary PCI, has been well established and occurs in a considerable number of patients with STEMI [89]. The two major pathological processes of microvascular injury that can be visualized and quantified by using CMR are called MVO and intramyocardial hemorrhage (IMH) [90]. Case examples of a STEMI patient without and with microvascular injury as visualized by CMR are shown in Figure 4.

MVO can be detected with several CMR techniques [91,92,93], but LGE is currently considered as the preferred methodology for assessing the presence and extent of MVO after STEMI [54]. At LGE, MVO is defined as a hypointense infarct core (dark) surrounded by the hyperintense infarct area (white). Most studies looking at the prognostic value of MVO after STEMI performed CMR between Days 3 and 7 after reperfusion therapy [24,94,95,96,97,98]. Although additional research focusing on the optimal post-STEMI timing for CMR performance is clearly needed, there is consensus that the above-mentioned time window seems most appropriate for comprehensive myocardial tissue characterization (including MVO assessment) by CMR after STEMI [54].

MVO is detected in approximately 50% of STEMI patients treated with primary PCI who undergo CMR within this time frame [94,95,99]. The presence and amount of MVO is associated with worse LV systolic and diastolic function, larger IS, diffuse tissue alterations in the non-infarcted myocardium, lack of functional recovery, and subsequent adverse remodeling [93,100,101,102,103,104,105,106,107]. A meta-analysis by Hamirani et al. confirmed the significant relation of MVO with worse systolic function, increased ventricular volumes, larger IS, and higher risk of adverse remodeling [94]. Importantly, the presence and extent of MVO not only plays a crucial role in infarct healing and adverse remodeling but it also entails a considerable higher risk for recurrent cardiovascular events and mortality. In fact, Hamirani et al. also identified seven studies including a total of 2132 patients that evaluated the impact of MVO on MACE [94]. In their meta-analysis, the presence of MVO was significantly associated with cardiac death, heart failure/heart failure hospitalizations and recurrent myocardial infarction. In line with these findings, a recent patient pooled analysis involving 1025 patients who underwent primary PCI and early CMR (within a median of four days) demonstrated that the presence of MVO was independently associated with MACE, defined as a composite of cardiac death, congestive heart failure, and re-infarction [95]. Interestingly, IS was not independently related with MACE in this analysis underscoring the recent notion that MVO may be an even stronger predictor of outcome than IS [24]. In a pooled analysis using individual patient data from seven randomized trials, de Waha et al. observed that every 1.0% absolute increase in MVO size was associated with a 14% and 8% relative increase in mortality and hospitalization for heart failure at one year, respectively [108]. This large study (*n* = 1688) confirmed the strong relation between MVO and hard clinical events even after adjustment for IS. Whether MVO can also predict long-term clinical outcomes beyond the first years after STEMI was evaluated only recently. Regenfus et al. showed that the extent of MVO was the strongest prognostic factor for six-year MACE rates in 245 STEMI patients [96]. Similar findings were reported from a multicenter registry including 810 revascularized STEMI patients [97]. MVO was associated with MACE in all multivariate models irrespective of whether it was entered as a dichotomous, continuous, or optimal cutoff variable [97]. In contrast, IS lost its prognostic relevance when corrected for MVO once again [97]. Furthermore, there was no significant interaction between IS and MVO on clinical outcome. These findings further underline the view that the prognostic significance of MVO cannot be explained due to the larger IS found in patients with MVO. In line with this concept, MVO significantly improved long-term risk stratification over traditional outcome predictors in both studies [96,97]. A recent study by Galea et al. specifically looked at the prognostic role of MVO in myocardial infarction patients with preserved ejection fraction (>50%) [98]. MVO provided prognostic information also in this subgroup of patients, further underscoring its role for optimized risk stratification after myocardial infarction. Taken together, there exists striking evidence supporting the use of MVO as determined by LGE-CMR in the early post-STEMI phase for improving short- and long-term risk stratification. Furthermore, CMR-detected LV ejection fraction, IS and MVO were integrated into a CMR score for risk stratification [109]. The score demonstrated good prediction of adverse outcome with incremental prognostic information over classic risk factors.

Parametric T1 mapping has been recently used for more sophisticated infarct core pathology evaluation by CMR. A prognosis study by Carrick et al. included 288 STEMI patients and found that infarct core pathology, defined by low infarct core native T1 values, was related with increased risk for poor outcome [110]. The authors consequently postulated that infarct core pathology by native T1 mapping might represent a new imaging biomarker with potential for prognostication after STEMI [110]. Further confirmatory studies are however needed to define the exact value of infarct core pathology, especially in comparison with MVO by LGE.

IMH is assumed to be a consequence of severe microvascular injury in the context of delayed reperfusion [99]. IMH can be depicted using several CMR methodologies [94], however T2* mapping is nowadays the sequence of choice [111,112,113]. Although closely linked to the presence of MVO, it was recently shown that IMH and MVO follow distinct time courses after primary PCI for acute STEMI [114,115]. In a serial imaging time-course analysis, IMH occurred in 23%, 43%, 33%, and 13% of patients 4–12 h, 2 days, 10 days, and 7 months after mechanical reperfusion [115]. Carrick and colleagues also observed that IMH was more consistently related with adverse remodeling and clinical events than MVO [115]. Most recently, the “Hemorrhage assessed by Cardiac Magnetic Resonance in ST-elevation Myocardial Infarction (HEM-CMR)” study confirmed that IMH was more closely associated with MACE than MVO in patients treated with primary PCI for acute STEMI [116]. Of note, IMH added incremental prognostic information to clinical risk factors and established CMR prognosis markers including IS and MVO.

In summary, CMR has greatly improved our understanding of the prognostic relevance of microvascular injury in the context of primary PCI for acute STEMI. The prognosis of STEMI patients worsens with the presence of MVO and is worst for those who also develop IMH. Therefore, assessment of microvascular injury (namely, MVO and IMH) should play a major role in CMR-based risk stratification soon after STEMI. The incremental value of IMH over MVO as well as the role of novel imaging biomarkers of infarct core pathology (e.g., native T1 mapping) needs further evaluation in dedicated studies. Beyond risk stratification, addressing MVO and IMH by novel therapeutic strategies may help to further improve clinical outcomes after PCI for acute STEMI. Thus far, however, efforts directed at ameliorating microvascular injury showed only very limited success [86,89].

## 6. Remote Myocardium

CMR imaging during the early phase after acute infarction is usually focused on myocardial function, infarct pathology, and infarct complications. However, besides the established role of CMR for assessment of LV function and characterization of the infarcted tissue, it recently evolved into a unique tool for evaluation of the non-infarcted myocardial tissue as well. In this regard, T1 mapping may be particularly useful to characterize abnormalities in myocardial tissue regions not directly affected by ischemia/reperfusion injury. Remote myocardium T1 values are significantly higher in patients with prior myocardial infarction compared to controls [117]. In addition, the severity of LV systolic dysfunction is independently related with T1 times derived from native T1 mapping. Higher native T1 values were further linked to higher C-reactive protein levels acutely and the presence of microvascular injury [118]. A small study involving 25 patients with acute infarction, 15 patients with chronic infarction and 20 controls, observed reduced systolic thickening and lower post-contrast myocardial T1 times indicative of extracellular matrix expansion in patients with acute myocardial infarction [119]. A natural history study by Carberry et al. included 140 STEMI patients who underwent CMR two days and six months after reperfusion therapy and showed that an increase in extracellular volume over time post-STEMI was correlated with an increase in LV end-diastolic volume [120]. Diffuse myocardial fibrosis, determined by native and/or post-contrast T1 mapping in the remote myocardium could therefore play an important pathophysiological role in LV dysfunction and remodeling after myocardial infarction. Importantly, T1 mapping is able to reveal subtle remote myocardium abnormalities, most likely representing diffuse fibrosis, not apparent on classical LGE sequences [121]. Carrick et al. evaluated the association between remote myocardium T1 values obtained by contrast-free T1 mapping and LV remodeling as well as adverse cardiac events in 267 STEMI patients [122]. Remote T1, assessed two days post-STEMI, was related with the change in LV end-diastolic volume from baseline to six months follow-up and the concentration of N-terminal pro-B-type natriuretic peptide levels at six months. Interestingly, increased native T1 was also associated with adverse cardiac events during a median follow-up of 845 days. In agreement with these findings, another T1 mapping study not only confirmed the prognostic importance of the tissue changes in the myocardial remote zone for recurrent cardiac events but also showed that the prognostic information of native T1 is independent and incremental to LV systolic function and infarct severity by LGE imaging [106]. Integration of CMR data on LV function, infarct pathology and remote alterations by native T1 yielded in optimized risk prediction in comparison with any one of the parameters in isolation. The above-mentioned observations indicate that not only the magnitude of infarct damage but also diffuse remote zone tissue alterations depicted by native T1 are of strong prognostic importance after STEMI. In this sense, a multi-parametric approach using CMR imaging for characterization of myocardial function, infarct damage, and remote zone alterations is likely most informative for patient-specific risk stratification of post-STEMI patients. In contrast with the promising data for native remote zone T1 mapping, the role of remote zone extracellular volume quantification using contrast enhanced T1 mapping for prediction of cardiovascular events remains to be determined.

## 7. Left Ventricular Thrombus

**Figure 5:**
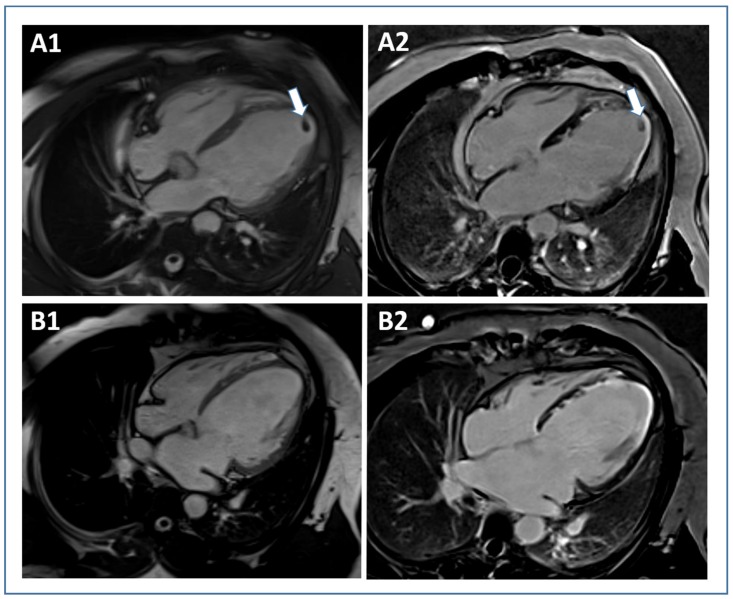
Left ventricular thrombus detection by cardiac magnetic resonance. Two representative patients with anterior STEMI: “Patient A” (first line) with left ventricular thrombus (white arrow, (**A1**,**A2**)); and “Patient B” without thrombus (**B1**,**B2**). Abbreviations: STEMI, ST-elevation myocardial infarction.

Formation of LV thrombus post-STEMI is well described and present in approximately 6% of patients undergoing CMR within the first week after the acute event [123]. The risk of LV thrombus is highest in patients with anterior infarction and reduced LV ejection fraction. In fact, in the subgroup with anterior STEMI and LV ejection fraction <50%, LV thrombus was described in one in five patients [123]. It is important to note that CMR imaging has a much higher sensitivity for LV thrombus detection when compared with echocardiography [123]. Case examples of patients with and without an LV thrombus are shown in Figure 5. In a multicenter CMR study involving 738 STEMI patients from eight centers, the presence of LV thrombus was associated with an increased rate of MACE at one-year follow-up [124]. In the same study, larger IS and increased extent of MVO by CMR were described as further predictors for the occurrence of LV thrombus [124]. The association between LV thrombi and MACE was independent of clinical variables (e.g., TIMI risk score) and IS. Nevertheless, presence of LV thrombus had no incremental prognostic value in addition to IS and MVO. As such, the higher risk of MACE in patients with LV thrombus is at least in part a reflection of more extensive myocardial infarction and MVO as well as worse cardiac function. Interestingly, an analysis of 1,035,888 STEMI patients from the Healthcare Cost and Utilization Project Nationwide Inpatient Sample showed a higher rate of in-hospital complications including in-hospital cardiac arrest and mortality [125]. LV thrombus was further associated with increased length of hospital stay and hospitalization charges [125]. Together, these data indicate that CMR is the optimal imaging tool to detect LV thrombi and that STEMI patients with LV thrombi should be regarded as high-risk patients.

## 8. Pericardial Injury

**Figure 6:**
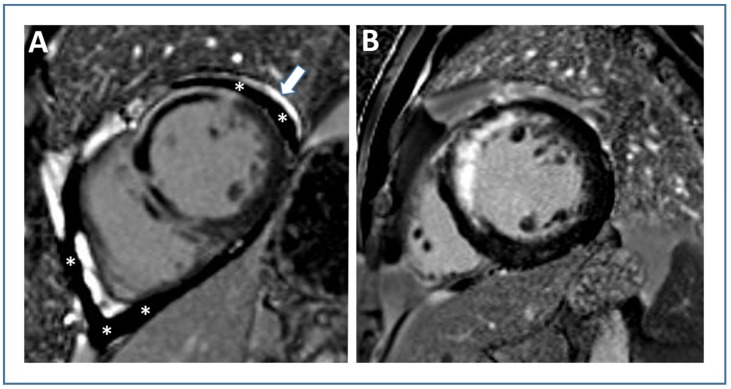
Pericardial injury due to acute STEMI. Late gadolinium-enhanced images of two patients with anterior wall STEMI: “Patient A” shows pericardial injury (pericardial effusion indicated by white stars, pericardial enhancement indicated by the white arrow, (**A**)), while no signs of pericardial injury are present in “Patient B” (**B**). Abbreviations: STEMI, ST-elevation myocardial infarction.

CMR imaging not only enables assessment of myocardial pathology but also accurately depicts early post-infarction pericardial injury (Figure 6). In a retrospective study involving 189 STEMI patients treated with primary PCI, Doulaptsis et al. found CMR evidence of pericardial injury in nearly half of patients [126]. It was defined as pericardial effusion on cine CMR and/or pericardial enhancement on LGE CMR [126]. Patients with pericardial injury had higher levels of cardiac troponin and C-reactive protein as well as more extensive myocardial and microvascular damage at CMR [126]. A further study evaluated the relationship between circulating C-reactive protein and the presence of pericardial injury as defined by pericardial effusion >4 mm and/or enhancement by LGE after myocardial infarction [127]. It could be shown that the rise in inflammatory biomarkers after infarction is not only due to myocardial injury but also due to pericardial injury, which in itself is an indirect sign of myocardial infarction severity [127,128]. These observations demonstrate that pericardial injury is common in the acute phase after STEMI and represents a marker of more extensive myocardial damage. The prognostic significance of pericardial injury, however, remains unknown from this study. Biere et al. reported on 193 first STEMI patients who underwent CMR within five days and three months after infarction [129]. The authors primarily aimed to identify determinants of pericardial effusion post-infarction. Systolic wall stress, IS and MVO extent emerged as independent predictors of pericardial effusion presence and volume in multivariable analysis [129]. In this study with a relative limited power, pericardial effusion did not seem to be associated with adverse clinical events [129]. The first and only multicenter CMR investigation looking at the prognostic significance of pericardial effusion was recently published by Jobs et al. [130]. This study looked for the presence of moderate-to-large pericardial effusion in 780 STEMI patients undergoing CMR imaging at median three days after infarction [130]. A quarter of patients exhibited a moderate-to-large pericardial effusion. These patients had more severe myocardial damage by CMR and worse LV function. Interestingly, the presence of moderate-to-severe pericardial effusion remained significantly associated with worse clinical outcome (all-cause death, reinfarction, and new congestive heart failure) within 12 months after STEMI even after adjustment for potential confounders [130]. Therefore, the presence of moderate-to-large pericardial effusion in the early phase after STEMI should be considered as a sign of more severe infarction with worse prognosis.

## 9. Aortic Stiffness

Aortic stiffening, which occurs in response to the cumulative exposure to hemodynamic loading, advancing age, and cardiovascular risk factors, has important implications for cardiovascular health [131]. It impacts the early systolic pressure rise, and thus myocardial wall stress is associated with a reduced coronary perfusion pressure, diastolic and systolic dysfunction, and adversely affects LV remodeling and myocardial fibrosis [131]. It is important to note that aortic stiffness improves prediction of cardiovascular events beyond conventional risk factors across multiple subpopulations [132].

Assessment of pulse wave velocity (PWV), defined as the ratio of the distance between two arterial sites and the transit time of the pulse between these sites, is considered as reliable and simple way to determine aortic stiffness in vivo. Carotid-femoral PWV is typically considered as the clinical reference standard for aortic stiffness assessment; however, several other methods exist [131]. Among them, the transit-time method using phase-contrast CMR emerged as robust variant to quantify PWV in vivo [133,134]. The feasibility and robustness of this approach has been successfully validated in patients with acute STEMI [135]. Although a large body of evidence has proven the prognostic implications of aortic stiffness in multiple populations, only few data exist for patients suffering a STEMI. Nevertheless, aortic stiffness has gained attention for risk prediction also in patients after STEMI [136,137]. Increased aortic stiffness as determined by aortic PWV using phase-contrast CMR has been shown to be correlated with elevated levels of natriuretic peptides and cardiac troponin in the acute and chronic stage after STEMI [138,139,140]. Imbalzano et al. evaluated the carotid-femoral pulse wave velocity in 136 STEMI patients [141]. Increased pulse wave velocity was independently associated with a less effective recovery of LV function at three and six months post-infarction. Similarly, Hirsch et al. described a significant relation between worse aortic stiffness and end-systolic volume index in patients after myocardial infarction [142]. A prospective observational study including 160 patients with first acute STEMI found that PWV measured two days after infarction using phase-contrast CMR is independently associated with an increased risk of major adverse cardiac and cerebrovascular events during a median follow-up of 1.2 years [137]. The assessment of aortic stiffness in addition to conventional risk factors significantly improved early risk classification in this study. Given the relatively low number of STEMI patients included in the above-mentioned investigations, a better understanding of the prognostic role of aortic stiffness in post-STEMI patients is still needed. Further studies should not only confirm the validity of these results but also address whether integration of aortic stiffness into an integrative CMR imaging prognosis model might have incremental predictive value for cardiovascular events post-STEMI.

## 10. Remaining Challenges and Opportunities

The timing of post-reperfusion CMR scans remains an important issue. Most CMR parameters of myocardial function and infarct severity change significantly over time [143,144,145,146]. Therefore, performing CMR very early after PCI (Day 1) leads to a considerable overestimation of IS because of edema and partial volume effect [147]. In contrast, IS by LGE and MVO are relative stable between Days 3 and 7 after STEMI and have strongest evidence for risk prediction in this time period [17,48,143]. From a clinical perspective, deferring the CMR scan to the subacute phase after STEMI (Day 3–7) might be reasonable for safety concerns as well. On the other hand, delayed risk stratification by deferring the scan >7 days (or even later) carries the risk of identifying high-risk patients too late. In this regard it is reassuring that a recent study by Masci et al. showed that early- (median four days), deferred- (median 4.8 months) or paired-CMR strategies were equivalent in predicting all-cause mortality and heart failure events over a median of 8.3 years [148]. We therefore believe that for a timely and efficient risk stratification after STEMI it is meaningful to aim for an early CMR strategy (Days 3–7) in the vast majority of cases.

In addition to the optimal timing of CMR after STEMI, there is also a great need for CMR protocol standardization [143]. For example, the impact of gadolinium dose and timing of LGE acquisition after contrast administration is well known [149,150]. Further efforts are thus needed to make CMR after STEMI more standardized.

Gadolinium-based contrast agents are considered safe and well tolerated in most patients with the exception of those with markedly reduced renal function. However, there is some recent data suggesting gadolinium-based contrast agent-related toxicity based on the accumulation of the contrast agent in multiple tissues, including bone, kidneys, and brain, despite intact renal function [151]. The clinical significance of gadolinium deposition remains to be determined [152]. Nevertheless, novel techniques that allow quantitative characterization of the infarcted and non-infarcted myocardium after myocardial infarction without the need of a gadolinium contrast agent have been investigated recently [97,153,154,155,156,157]. CMR infarct characterization and prognostication without using contrast agents is an interesting concept, but the prognostic role of non-contrast CMR compared to contrast-enhanced CMR remains to be defined.

Incorporating CMR scans into clinical routine is difficult because of several reasons including the low availability and relative high costs of CMR as well as logistic concerns. Simple, fast, and robust protocols allowing high quality CMR infarct characterization are required to make CMR more feasible in daily clinical routine. Although there have been progresses in accelerating the acquisition of CMR, validation and prognostic evaluation of such protocols are currently lacking [3,11,69,158]. Even more important, there are thus far no data from prospective randomized controlled trials showing that CMR-guided risk stratification improves patient outcome. There is therefore an unmet need for well-designed trials that investigate if imaging with CMR to depict infarct pathology soon after STEMI is able to further improve outcomes in this group of patients. Such studies should also incorporate an economic evaluation based on cost-effectiveness analysis.

Although evidence for CMR risk prediction mainly comes from the recent era of current “state-of-the-art” STEMI management, the impact of novel post-STEMI treatment strategies (e.g., novel medications) on the predictability of CMR needs continuous evaluation.

## 11. Summary

**Figure 7:**
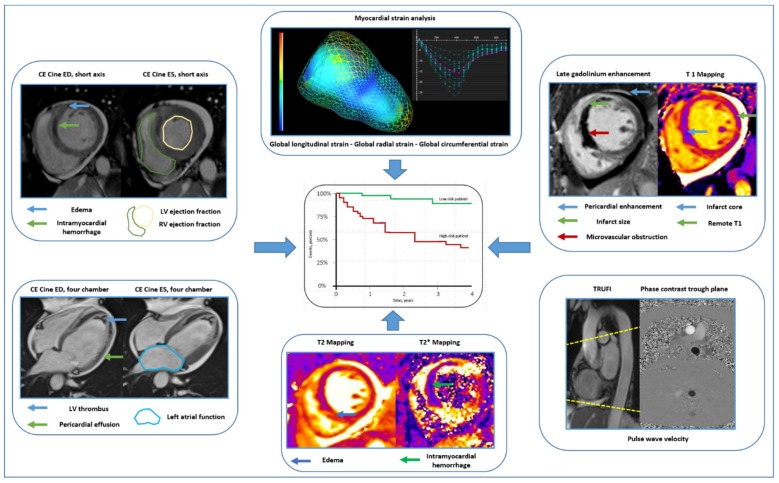
Cardiac magnetic resonance to improve risk prediction after ST-elevation myocardial infarction. Exemplary illustration of comprehensive characterization of myocardial function and tissue damage soon after ST-elevation myocardial infarction using contemporary CMR imaging. Abbreviations: CE, contrast enhanced; ED, end-diastolic; ES, end-systolic; LV, left ventricular; RV, right ventricular.

Imaging plays a central role in early risk stratification after acute STEMI. In current clinical routine, LV ejection fraction is still the most relevant predictor of adverse outcome post-STEMI and forms the basis for several treatment decisions. CMR, however, not only affords a highly accurate characterization of cardiac function (and therefore ejection fraction), but it also provides exact depiction of myocardial injury (Figure 7). In this sense, IS and MVO by LGE CMR emerged as robust outcome predictors with incremental prognostic information in addition to clinical and angiographic risk factors as well as LV ejection fraction. For predicting the risk of short- and long-term outcomes, risk assessment using CMR as early as possible is desirable. It is therefore reassuring that recent evidence shows that CMR early after reperfusion is equally effective in predicting outcome when compared with CMR performed after the subacute phase of STEMI. Pragmatic CMR scores for optimized stratification of risk soon after STEMI have been developed recently. These scores have integrated traditional CMR information on myocardial function (LV ejection fraction) and infarct pathology (IS and MVO) and might further simplify the use of CMR in the clinical setting. However, there is very recent evidence for the incremental prognostic value of more advanced markers of myocardial function (mostly myocardial strain) and myocardial tissue pathology (mainly IMH and native T1 of the non-infarcted myocardial tissue) after STEMI. The exact role of these novel CMR tissue biomarkers in comparison to traditional CMR outcome markers remains to be defined. A further drawback for the clinical application of CMR as risk stratification tool post-STEMI is the low availability of CMR in clinical routine as well as the lack of standardization in timing the post-reperfusion scan, imaging protocols, and post-processing. The recent JACC scientific expert panel consensus document for using CMR endpoints in myocardial infarction trials might serve as a guide in this sense. Another relevant limitation is that there are currently no established therapeutic approaches for targeting abnormalities in myocardial and microvascular tissue as depicted by CMR.

Together, CMR has great potential for improving patient-specific risk stratification soon after primary PCI for STEMI. An integrative CMR risk score including information on myocardial function and myocardial tissue pathology is likely the most informative for stratifying risk post-STEMI. Additional research is, however, necessary before using CMR as risk and treatment stratification tool in daily clinical routine.

## Abbreviations

AAR	Area at Risk
CMR	Cardiac Magnetic Resonance
HEM-CMR	Hemorrhage Assessed by Cardiac Magnetic Resonance in ST-elevation Myocardial Infarction
IMH	Intramyocardial Hemorrhage
IS	Infarct Size
JACC	Journal of the American College of Cardiology
LA	Left Atrial
LGE	Late Gadolinium Enhancement
LV	Left Ventricular
MACE	Major Adverse Cardiovascular Events
MVO	Microvascular Obstruction
PCI	Percutaneous Coronary Intervention
PWV	Pulse Wave Velocity
PSIR	Phase-sensitive Inversion Recovery
RV	Right Ventricular
STEMI	ST-elevation Myocardial Infarction
STIR	Short Tau Inversion Recovery
SSFP	Steady-state Free Precession
TIMI	Thrombolysis in Myocardial Infarction
